# MicroRNA Profiling and Head and Neck Cancer

**DOI:** 10.1155/2009/837514

**Published:** 2009-06-01

**Authors:** Xiqiang Liu, Zugen Chen, Jinsheng Yu, James Xia, Xiaofeng Zhou

**Affiliations:** ^1^Center for Molecular Biology of Oral Diseases, UIC Cancer Center, College of Dentistry, Graduate College, University of Illinois at Chicago, Chicago, IL 60612, USA; ^2^Guanghua School & Research Institute of Stomatology, Sun Yat-Sen University, Guangzhou 510055, China; ^3^Department of Human Genetics & UCLA Microarray Core Facility, University of California at Los Angeles, Los Angeles, CA 90095, USA; ^4^GenoSensor Corporation, Tempe, AZ 85282, USA

## Abstract

Head and neck/oral cancer (HNOC) is a devastating disease. Despite advances in diagnosis and treatment, mortality rates have not improved significantly over the past three decades. Improvement in patient survival requires a better understanding of the disease progression so that HNOC can be detected early in the disease process and targeted therapeutic interventions can be deployed. Accumulating evidence suggests that microRNAs play important roles in many human cancers. They are pivotal regulators of diverse cellular processes including proliferation, differentiation, apoptosis, survival, motility, and morphogenesis. MicroRNA expression patterns may become powerful biomarkers for diagnosis and prognosis of HNOC. In addition, microRNA therapy could be a novel strategy for HNOC prevention and therapeutics. Recent advances in microRNA expression profiling have led to a better understanding of the cancer pathogenesis. In this review, we will survey recent technological advances in microRNA profiling and their applications in defining microRNA markers/targets for cancer prediction, diagnostics, treatment, and prognostics. MicroRNA alterations that consistently identified in HNOC will be discussed, such as upregulation of miR-21, miR-31, miR-155, and downregulation of miR-26b, miR-107, miR-133b, miR-138, and miR-139.

## 1. Introduction

### 1.1. Head and Neck/Oral Cancer

Head and neck/oral cancer (HNOC) is the sixth most common cancer worldwide accounting for 4% of cancers in men and 2% of cancers in women [1]. In some parts of the world, including Southern China and the Indian subcontinent, HNOC is a major cancer problem. In the US, one patient dies from HNOC every hour. HNOC is one of the undertreated and understudied diseases. According to American Cancer Society—Cancer Statistics [2–6], the new cases for HNOC increased approximately 25% during the past 5 years, where the overall new cancer cases only increased about 5% in the same period (Table 1). More strikingly, the death associated with HNOC increased by 5% during the past 5 years, while death associated with all cancers stayed the same. This represents a major health problem, and may be linked to suboptimal treatment outcomes and clinical decision making.

Over 90% of HNOC are oral squamous cell carcinoma (OSCC), carcinomas arising from the epithelium lining of the oral cavity. The initiation and progression of OSCC is a complex multistep process that entails a progressive acquisition of genetic and epigenetic alterations. HNOC has traditionally been causally associated with heavy smoking and alcohol abuse [7]. Of these, tobacco smoking is well established as a dominant risk factor for OSCC, and this risk is correlated with the intensity and duration of smoking. Nevertheless, the increased incidence of HNOC in nonsmokers and nondrinking patients in recent years suggests that other environmental, immunologic, or genetic factors also contribute to the pathogenesis of HNOC [8]. Human papillomavirus (HPV) is the major etiologic factor in the development of cervical cancer, and has been studied extensively [9]. Results of recent molecular and epidemiologic studies suggested that HPV is also an important etiologic factor in a subset of HNOC [10], particularly those that developed at pharynx sites, such as oropharyngeal and tonsillar cancers. According to the American Cancer Society: Cancer Statistics [2–6], the new cases for pharynx cancer increased over 50% during for the past 5 years, where the over all new cancer cases only increased about 5% in the same period (Table 1).

### 1.2. Microrna Biogenesis and Functions

Like most of the other human cancers, HNOC is a disease involving multistep dynamic changes in the genome. However, most studies on the cancer genome have focused most heavily on protein-coding genes, and our knowledge of alterations of the noncoding sequences in cancer is largely absent. In the past several years, the biomedical fields have manifested a rapidly expanding interest on a relatively small number of small genes—microRNAs (miRNAs). MicroRNAs are newly recognized, noncoding, regulatory RNA molecules, about 22 nucleotides (nts) in length, and found in all metazoans studied thus far. It is estimated that the human genome may have 800–1000 microRNAs [11]. Although they account for only a very minor fraction of the expressed genome, microRNAs are pivotal regulators of development and cellular homeostasis through their control of diverse cellular processes including proliferation, differentiation, apoptosis, survival, motility, and morphogenesis. 

The microRNA biogenesis has been investigated extensively. While most of the microRNAs are transcribed by the RNA polymerase II to produce a primary-microRNA (pri-microRNA), approximately 20% of human microRNAs are transcribed by RNA polymerase III. Pri-microRNAs are usually long nucleotide sequences, and some of them have several hundreds to a thousand nucleotides. The pri-microRNA is spliced and usually capped with a 5′ 7-methylguanosine cap (^m7^G) and poly-adenylated at the 3′ end, similar to protein-coding mRNAs. Then, pri-microRNAs form specific hairpin-shaped stem-loop secondary structures and enter a microprocessor complex (500–650 kDa) consisting of a Drosha (a RNase III endonuclease) and an essential cofactor DGCR8/Pasha (protein containing two double-stranded RNA binding domains). There they are processed into a 60- to 70-nt pre-microRNA with a 5′ phosphate and a 3′ 2 nt overhang. The pre-microRNAs are then transported to the cytoplasm by Exportin-5 (Exp5) (a member of the Ran transport receptor family). Once in the cytoplasm, pre-microRNAs are further processed to a short double strand microRNA:microRNA* duplex by Dicer, a second RNase III endonuclease. Finally, the microRNA:microRNA* duplex is unwound into a mature microRNA and microRNA* by a helicase. The mature microRNAs are asymmetrically incorporated into the RNA-induced silencing complex (RISC) where they regulate gene expression by mRNA degradation or translational repression while the microRNA* is quickly degraded.

**Figure 1:**
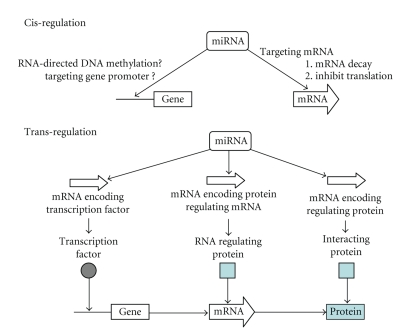
*Potential microRNA regulation mechanisms.* The potential mechanisms of microRNA-mediated gene regulation are multifactorial and encompass interaction(s) among different mechanisms. *Cis-regulation:* microRNA direct targeting the mRNA and regulating the expression of the target gene at post-transcriptional levels (e.g., enhance mRNA degradation and inhibit translation). It has also been suggested that microRNA can control gene transcription based on potential mechanisms. *Transregulation:* Following the expression changes of microRNA targeted specific genes (e.g., genes coded for transcription factors, genes coded for RNA regulating proteins, and genes coded for proteins that will interact with the target protein), subsequent effects may alter the transcription of other gene, levels of other mRNAs, or interactions among proteins, and thus microRNA may exert its functional effects through transregulatory mechanism(s).

However, microRNAs are not involved directly in protein coding, but are believed to control the expression of more than one-third of the protein-coding genes in the human genome [12–14]. Each microRNA can target and regulate the mRNA transcripts of hundreds of genes downstream. One microRNA can have multiple target sites in the mRNA transcript of a downstream gene, while one mRNA can be targeted by multiple microRNAs. Therefore, microRNAs contribute a newly recognized level of regulation of gene expression. As illustrated in Figure 1, the potential mechanisms of microRNA-mediated gene regulation are multifactorial and encompass interaction(s) among different mechanisms. It has been demonstrated that microRNA direct-target the mRNA and regulate the expression of the target gene at post-transcriptional levels (e.g., enhance mRNA degradation and inhibit translation). This cis-regulation occurs by binding of the ~21 nucleotide mature microRNA to an imperfectly matched sequence in the target mRNA. Following the expression changes of specific microRNA-targeted genes (e.g., genes code for transcription factors, and genes code for RNA regulating proteins), subsequent effects may alter the levels of other mRNAs (or protein interaction), and thus microRNA may exert its effects on the expressed genome through transregulatory mechanism(s). 

For more details on microRNA biogenesis, basic functions, and their roles in normal physiology and diseases, numerous excellent reviews are recommended [15–21]. In the following sections, we survey recent technological advances in microRNA profiling of tumor cells and consider some of the major challenges that remain in defining microRNA markers/targets for cancer prediction, diagnostics, treatment and prognostics. We will also identify potential solutions that may overcome these outstanding challenges to improve the diagnosis and treatment of cancer.

## 2. Overview of the Commonly Used MicroRNA Profiling Technologies in HNOC Studies

High-throughput microRNA gene expression analysis is a technical challenge. The short length and uniqueness of each microRNA render many conventional tools ineffective—very small RNAs are difficult to reliably amplify or label without introducing bias. Earlier attempts for detection and identification of microRNA included 3 approaches: hybridization-based methods (e.g., Northern blot), PCR-based detection, and cloning methods. Based on these initial approaches, higher-throughput technologies have been developed to systematically profile microRNA at genome-wide scale. Here, we provide a review of these technologies and their utilization in HNOC research (Table 2). 

### 2.1. Hybridization-Based Microrna Profiling—Microarray

The hybridization-based microRNA detection methods include Northern blotting, in situ hybridization, bead-based flow-cytometry, and microarray. The majority of the published studies reporting microRNA profiling analysis were performed using different microarray technologies. The differences in these microarray platforms are mainly in their probe design, probe immobilization chemistry, sample labeling, and signal detection methods (see [28] for comprehensive review on array-based microRNA profiling). Typical to the evolution of mRNA microarray platforms during the early stage of microRNA array development, most of the arrays were custom made. For example, using a custom microRNA microarray [29], Tran et al. presented the first microarray-based microRNA profile of HNOC using 9 HNOC cell lines [22]. Thirty-three microRNAs in the array were found to be highly expressed (including let7a, miR-16, miR-21, and miR-205) and 22 showed low levels of expression (including miR-342, miR-346, and miR-373*) in all cell lines. However, the drawback of this study is the lack of inclusion of normal control cell lines, which hinders the ability to detect relative difference in specific microRNA levels between normal and diseased cells. Nevertheless, this study provides the largest genome-wide survey of mature microRNA transcripts in head and neck cancer cell lines at the time. 

With the introduction of several commercially available microRNA array platforms, the study design and data analysis became more streamlined. Using GenoExplorerTM microRNA array from GenoSensor Corporation (Tempe, Ariz, USA), which contains 646 mature and pre-microRNAs, Chang et al. screened for altered microRNA expression in both HNOC primary tissue samples, HNOC cell lines, and normal control samples [24]. Eight microRNAs were found by to be upregulated (including mir-21, let-7, 18, 29c, 142-3p, 155, 146b) and one downregulated (miR-494) in cancer. Another example of using commercially available platforms for profiling study was designed to define the HNOC cell microRNA expression pattern under hypoxia condition [23]. The Human_V7.1C_051017 miRNA array (LC Sciences) was used in this study, which consists of approximately 260 microRNAs that were deposited in miRBase Version 7.1 (Sanger Institute, UK) at that time. The latest version of human microRNA array from LC Sciences consists of 856 microRNA (corresponding to Sanger miRBase Version 12.0). Nevertheless, this study identified 20 upregulated and 16 downregulated microRNAs in HNOC cells cultured under hypoxia condition [23]. 

While the currently available commercial microRNA arrays make profiling studies on microRNA a lot easier for biomedical research laboratories, several new developments in the biotech field have emerged as potential opportunities to improve microRNA microarrays. Locked nucleic acid (LNA) has quickly gain popularity in various biological and biomedical fields in the recent years due to its unprecedented affinity and specificity to the complementary RNA. LNA is a conformational RNA analogue that contains at least one LNA monomer. The unique feature of the unprecedented thermal stability between LNAs and their target RNA molecules enables efficient visualization of microRNA for in situ hybridization. In addition, the high metabolic stability of LNA along with enhanced microRNA recognition properties suggest that LNA-antimiRs could be an important tool for novel therapeutic approaches based on cancer-associated microRNAs. Attempts have also been made to incorporate LNA-based probes in the design of microarrays for microRNA profiling [30–32], which appears to also improve the mismatch discrimination. While it has not been utilized in HNOC studies, this platform has recently been used in studying several other malignancies, including chronic myeloid leukemia and breast cancer [33, 34]. A recent review by Stenvang et al. provided a comprehensive review on recent progress in LNA-based microRNA detection in cancer [35]. 

Other attempts to improve the microarray-based microRNA profiling include the introduction of the RNA-primed array-based Klenow enzyme (RAKE) assay [36] and the development of modified RAKE assays [37]. The RAKE assays are based on the ability of an RNA molecule to function as a primer for Klenow polymerase extension when fully base-paired with a single-stranded DNA molecule. When combined with microarray technology, RAKE appears to provide better specificity than other microarray platforms. It has also been reported that with this RAKE assay, microRNAs isolated from formalin-fixed paraffin-embedded tissue can be utilized to generate optimal quality microRNA profiles [36, 38], which opens up new opportunities for analyses of small RNAs from archival human tissue.

### 2.2. QRT-PCR-Based MicroRNA Profiling

While the microarray-based profiling methods described earlier have excellent throughput and high coverage, these methods do not amplify the microRNA and thus often compromise the sensitivity. Real-time quantitative PCR-based approaches have unparalleled sensitivity and specificity. However, it is technically challenging to amplify and quantify mature microRNA because the mature microRNA is only around 22 nts, roughly the size of a standard PCR primer. Therefore, earlier versions of qRT-PCR-based method are usually quantifying microRNA precursors. For example, a large panel of TaqMan assays were designed for 222 hairpin-containing microRNA precursors based on RT and PCR amplification using gene-specific primers, and quantification using TaqMan minor groove binder (MGB) probes that are specific to the loop portion of the microRNA precursor [39]. Using this TaqMan assay panel, the microRNA precursor expression was profiled in 32 human cancer cell lines, including 5 HNOC cell lines [39]. Interestingly, unsupervised clustering analysis based on the expression values of these 222 microRNA precursors was able to cluster most of the cancer cell lines into tissue-specific groups. This suggested that the existence of a tissue-specific microRNA expression signature for cancers originated from various tissues. It should be emphasized that this profile is for the microRNA precursors and not the active, mature microRNA. While the relative level of most mature microRNAs may be predicted based on the level of corresponding precursors, additional tests will be needed to ensure that the expression of the mature microRNA is reflected by the precursor expression.

Recently a second generation of TaqMan microRNA assay has be developed to directly quantify the mature microRNA. This assay incorporates a target-specific stem-loop, reverse transcription primer. The innovative design addresses a fundamental challenge in microRNA quantification: the short length of mature microRNAs (~22 nt) prohibits conventional design of a specific quantitative real-time PCR assay. The stem-loop structure provides specificity for only the mature microRNA target and forms an RT primer/mature microRNA-chimera that extends the 3′ end of the microRNA. The resulting longer RT product presents a template amenable to standard real-time PCR using TaqMan Assays. These latest qPCR assays are commercially available (e.g., TaqMan MicroRNA Assay from Applied Biosystems). These assays can be packed into a convenient, preconfigured micro fluidic card that contains up to 384 unique TaqMan assays, and compatible with most of the commonly used qPCR instruments. Using this new TaqMan assay, the microRNA alterations for HNOC were identified using HNOC cell lines (compared to the immortalized human oral keratinocyte line) [27], and laser microdissected cancer cells from primary HNOC of tongue (compared to the paired normal tissues) [25]. 

### 2.3. Cloning and Sequencing Based MicroRNA Profiling

This approach is developed by combining aspects of direct microRNA cloning and serial analysis of gene expression (SAGE) technology, which lead to its name miRAGE [40]. Similar to traditional cloning approaches, miRAGE starts with the isolation of 18- to 26-base RNA molecules to which specialized linkers are ligated, and which are reverse-transcribed into cDNA. However, subsequent steps, including amplification of the complex mixture of cDNAs using PCR, tag purification, concatenation, cloning, and sequencing, have been performed by using SAGE methodology optimized for small RNA species. Using this technique, Cummins et al. were able to perform a large scale experimental analysis of microRNAs in human colorectal cells [40]. Sequence analysis of 273.966 small RNA tags led to identification of 200 known mature microRNAs, 133 novel microRNA candidates, and 112 previously uncharacterized microRNA* forms. 

SAGE was originally designed to characterize gene expression profiles. It has a potential to be a high-throughput gene expression profiling tool. Over the years, much improvement has been made to increase sequencing efficiency and reduce input RNA amount requirement [41–46]. Although it is not as popular as microarrays and qRT-PCR due to technological and economical challenges, this technology has the unique advantage of combining discovery and quantification.

## 3. Identified MicroRNA Players in HNOC

The elucidation of how global microRNA expression contributes to phenotypic outcomes in cancer is critically important. On the other hand, it is equally important to define the signaling pathways, as well as environmental or genetic factors that affect microRNA expression. Currently, most of the microRNA-related studies on cancer are based on the different expression profiling of microRNAs, which lead to the identification of many so-called “cancerous” microRNAs. Recent studies have suggested that microRNA signatures can be used to classify human cancers with accuracy compatible to mRNA signatures [47, 48]. Knockdown or overexpression of a specific microRNA allows functional validation and investigation of the specific roles of the microRNA in tumorigenesis. MicroRNAs have been functionally classified as proto-oncogenes or tumor suppressors and their aberrant expression has been reported in many cancer types including bladder cancers [49], breast cancer [50–52], cervical cancer [53–57], colorectal cancer [58–60], leukemia [61, 62], liver cancer [63–65], lung cancer [66–68], lymphoma [69], pancreatic tumors [70], and thyroid cancer [71]. Dysregulation (e.g., overexpression or loss of expression) of these “cancerous” microRNAs can contribute prominently in tumor initiation and progression by elaborating an inappropriate cellular program that promotes uncontrolled proliferation, that favors survival, that inhibits differentiation and/or promotes invasive behavior [72, 73]. 

As illustrated in Table 2, a number of studies have been recently carried out aiming for the identification of specific microRNA alterations in HNOC [22–25, 27]. Among those microRNAs identified, 6 of them have been functionally tested in HNOC to demonstrate their “cancerous” functions. These include miR-21, miR-184, miR-133a/133b, miR-137, and miR-193a. MiR-21 is a well-established oncogenic microRNA which enhances the cell proliferation and suppresses apoptosis [24, 74–77]. The upregulation of miR-21 has been observed in both HNOC tissue samples and HNOC cell lines [24, 25], as well as many other cancer types. Functional tests in HNOC cell lines confirmed that miR-21 enhances the cell proliferation and suppresses apoptosis [24]. The knowledge on miR-184 and its role on tumorigenesis are still elusive. Wong et al. suggested that miR-184 acts as a oncogenic microRNA by inhibiting apoptosis and inducing proliferation in HNOC [25]. However, a tumor suppressor function was suggested by a recent study showing that ectopic expression of miR-184 suppresses Akt signaling pathway, which is associated with a marked increase in keratinocyte apoptosis and cell death [78]. More studies will be needed to resolve this apparent contradiction. While miR-133a downregulation in HNOC has been reported by Wong et al. [25], downregulation of miR-133b has been consistently observed by both Wong and Kozaki studies [25, 27]. The downregulation of miR-133b has also been observed in colorectal cancer [59]. In a followup study, Wong et al. further demonstrated the tumor-suppresser functions of miR-133a/133b that inhibit proliferation and induce apoptosis in HNOC cell lines [26]. In addition to their apparent tumor-suppresser function, miR133a/133b have also been associated with various functional roles in cardiomyocytes [79–81], osteoblasts [82], and neurons [83]. It has been suggested that both miR-137 and miR-193a are silenced by DNA hypermethylation in HNOC [27]. This epigenetic event has also been observed in glioblastoma for silencing miR-137 [84]. The ectopic transfection of miR-137 or miR-193a into OSCC lines lacking their expressions significantly reduced cell growth, with downregulation of the translation of cyclin-dependent kinase 6 or E2F transcription factor 6, respectively [27]. These observations are consistent with previous findings in which tumor-suppressing roles have been suggested for miR-137 in glioblastoma [84] and melanoma [85], and for miR-193a in hepatocarcinoma, lung epithelial carcinoma, and cervical adenocarcinoma cell lines [86]. Another functionally validated microRNA in HNOC is miR-98, which is upregulated under hypoxia condition [23]. Transfection of pre-miR-98 diminishes high mobility group A2 (HMGA2) and potentiates resistance to chemotherapeutic drugs, doxorubicin, and cisplatin [23]. These findings suggest that miR-98 serves as a key element in modulating tumors in variable microenvironments. 

In addition to those functionally validated microRNAs described earlier, upregulations of miR-155 and miR-31 in HNOC have been observed in at least 2 independent studies we reviewed. MiR-155 has been suggested previously as an oncogenic microRNA [77]. MiR-155 has also been shown to infer mammalian innate and adaptive immunity, and viral infection [87]. The expression of miR-155 is regulated by transforming growth factor beta/Smad pathway [88], which is frequently elevated in HNOC [89]. While miR-31 upregulation has been consistently observed in HNOC [25, 27], its role in tumorigenesis is not entirely clear. The upregulation of miR-31 has also been observed in colorectal cancer [59, 60], hepatocellular carcinoma [65], but reduced expression of miR-31 was observed in breast cancer [52], and frequent homozygous deletion of miR-31 gene was reported in urothelial carcinomas [90]. 

Also, downregulation of several microRNAs has been consistently observed in HNOC, including miR-26b, miR-138, miR-107, miR-139. The role of miR-26b in cancer is not clear. While downregulation of miR-26b has been observed in HNOC [25, 27], upregulation of miR-26b has been observed in bladder cancers [49], and polycythemia vera platelets [91]. Interestingly, miR-26 family members have been shown to be induced by hypoxia [92] and downregulated by exposure to cigarette smoke [93]. It is worth knowing that Myc oncogene suppresses miR-26a, another member of the miR-26 family, which influences cell cycle progression by targeting the oncogene EZH2 in a murine lymphoma model [94]. The observation of downregulation of miR-138 in HNOC is consistent with a similar observation in thyroid cancer, in which the downregulation of miR-138 has also been associated with enhanced telomerase reverse transcriptase (TERT) expression [71]. In addition, miR-138 appears to play an important role in embryonic development, as it is required for cardiac morphogenesis during embryonic development in a temporal knockdown zebrafish model [95]. The observed downregulation of miR-107 in HNOC is consistent with its previously suggested tumor suppresser function in lung carcinoma cells [68]. However, significant overexpression of miR-107 (and its highly homologous miR-103) has been observed in pancreatic tumors [70]. High expression of miR-103/107 was also correlated with poor survival for patients with esophageal squamous cell carcinoma [96]. Other interesting observations include that of downregulation of miR-107 is specifically associated with HNF1alpha in hepatocellular tumors [97]. Also, all-trans-retinoic acid (ATRA) treatment lead to upregulated miR-107 in acute promyelocytic leukemia cells [98]. While downregulation of miR-139 has been observed in HNOC [25, 27], relative little is known of the relationship of miR-139 and cancer [99]. More studies will be needed to define the role of miR-139 in tumorigenesis. 

Recently, attempts have also been made to translate these identified microRNA alterations in HNOC to clinical utilities, including a preliminary study testing the feasibility of using miR-205 as a marker to detect metastatic HNOC cells in lymphoid tissues [100]. The association between high miR-211 level and poor prognosis of HNOC has also been suggested [101]. These data lay the foundation for additional studies that use microRNA to improve the diagnosis, prognosis, and staging of HNOC. 

## 4. Conclusion and Future Directions

Despite advances in diagnosis and treatment, mortality rates of HNOC have not improved significantly over the past three decades, which points to the immediate need for a better understanding of this disease. Accumulating evidence suggests that microRNAs play important roles in many human cancers, including HNOC. They are pivotal regulators of diverse cellular processes including proliferation, differentiation, apoptosis, survival, motility, and morphogenesis. Recent advances in microRNA expression profiling have led to a better understanding of the HNOC pathogenesis. This will lead to the identification of specific microRNA expression patterns that may become powerful biomarkers for diagnosis and prognosis of HNOC. In addition, these microRNAs may also serve as therapeutic targets for novel strategies of HNOC prevention and therapeutics. 

It is important to notice that HNOCs are groups of diverse cancers that develop from many different anatomic sites and are associated with different risk factors [102], genetic characteristics [103], and different clinical outcomes [104, 105]. Currently, most of the existing microRNA profiling studies on HNOC include cases from multiple anatomic sites. It will be ideal to obtain the site-specific microRNA signatures for various HNOCs, which should lead to substantial translational outcomes that will advance the management of these diverse HNOC types. This has been realized and a few studies have been devoted to the identification of site-specific microRNA signature for HNOC, including a study by Wong et al. 2008, specific microRNA profiles were defined for the SCC of tongue [25], one of the more aggressive type of HNOC in terms of invasion, spread, and recurrence [106, 107].

## Figures and Tables

**Figure 2:**
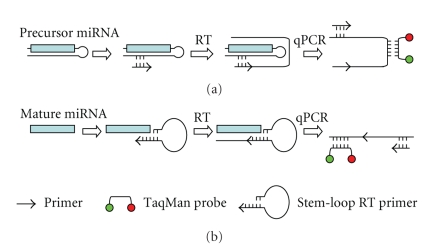
*TaqMan assays for quantifying precursor microRNA and mature microRNA*. (a) TaqMan assay designed to quantify hairpin-containing microRNA precursors. RT reaction and PCR amplification are performed using gene-specific primers. Quantification is carried out using TaqMan minor groove binder (MGB) probes that are specific to the loop portion of the microRNA precursor. (b) TaqMan assay designed to quantify mature microRNA. This assay utilizes a target specific stem-loop, reverse transcription primer for RT reaction. The stem-loop structure provides specificity for only the mature microRNA target and forms a RT primer/mature microRNA-chimera that extends the 3′ end of the microRNA. The resulting RT product will be quantified using standard TaqMan quantitative real-time PCR method.

**Table 1:** The changes of head and neck cancer incidence and death for the past 5 years. (Based on ACS: Cancer Statistics 2004, 2005, 2006, 2007, 2008 [2–6]).

Year	New Cases			Deaths		
	All cancers	Head and neck cancer	Pharynx cancer	All cancers	Head and neck cancer	Pharynx cancer

2004	1,368,030	28,260	8,250	563,700	7,230	2,070
2005	1,372,910	29,370	8,590	570,280	7,320	2,130
2006	1,399,790	30,990	8,950	564,830	7,430	2,110
2007	1,444,920	34,360	11,800	559,650	7,550	2,180
2008	1,437,180	35,310	12,410	565,650	7,590	2,200
5-yr total	7,022,830	158,290	50,000	2,824,110	37,120	10,690

5-year increase	69,150	7,050	4,160	1,950	360	130
Percent increase	5.1%	24.9%	50.4%	0.3%	5.0%	6.3%

**Table 2:** Recently identified microRNA alterations in HNOC.

Profiling study	Methods	microRNA alterations	Functional validation
Tran et al., 2007 [22] Profiling for 261 miRNAs was carried out on 9 HNOC cell lines. No normal control was included in this study.	Microarray	High expression in HNOC: miR-21, miR-23a, let-7f, miR-205, miR-31, let-7d, miR-221, let-7a, miR-320, miR-23b, miR-24, let-7c, miR-29b, miR-30b, miR-15a, miR-22, miR-107, miR-200b, miR-18, miR-16, miR-15b, miR-200a, miR-27a, let-7b, miR-28, hcv-miR-US33-1, miR-100, miR-98, miR-103, miR-125b, miR-361, miR-19a	
		Low expression in HNOC: has-miR-345, miR-449, miR-302b, miR-382, miR-373, miR-378, miR-200c, miR-340, miR-302c, miR-154, miR-371, miR-127, miR-133a, miR-302d, miR-328, miR-212, miR-375, miR-373*, hcv-miR-US25-2-5p, miR-133b, miR-346, miR-342	
			
Hebert et al., 2007 [23] Profiling for ~260 miRNAs was carried out on 3 HNOC cell linescultured under normoxic (5%O_2_) or hypoxic conditions (1%O_2_).	Microarray	Induced by hypoxia: miR-572, miR-214, miR-563, miR-637, miR-98, miR-628, miR-191, miR-210, miR-31, miR-498, miR-373, miR-19a, miR-148a, miR-15a, miR-200a, miR-7, miR-30b, let-7e, let-7g, let-7i	miR-98: regulates HMGA2 expression and chemosensitivity to doxorubicin and cisplatin.
		Suppressed by hypoxia: miR-122a, miR-565, miR-195, miR-30e-5p, miR-374, miR-19a, miR-101, miR-424, miR-186, miR-29b, miR-148b, miR-141, miR-22, miR-331, miR-422b, miR-197	
			
Chang et al., 2008 [24] Profiling for 314 miRNAs was carried out on 4 HNOC tissue samples (with 4 normal tissues as control). Concurrently, 4 HNOC cell lines and a normal oral keratinocyte cell line were also profiled.	Microarray	Upregulated in HNOC: **miR-21**, let-7, miR-18, miR-29c, miR-142-3p, **miR-155**, miR-146b	miR-21: regulates cell growth, cytochrome C release, and apoptosis.
		Downregulated in HNOC: miR-494	
			
Wong et al., 2008 [25, 26] Profiling for 156 miRNAs was carried out on 4 HNOC of tongue and 4 paired normal tissues.	qRT PCR	Upregulated in HNOC: miR-184, miR-34c, miR-137, miR-372, miR-124a, **miR-21**, miR-124b, **miR-31**, miR-128a, miR-34b, miR-154, miR-197, miR-132, miR-147, miR-325, miR-181c, 198, **miR-155**, miR-30a-3p, miR-338, miR-17-5p, miR-104, miR-134, miR-213.	miR-184: regulates proliferation, c-Myc expression, and apoptosis. miR-133a/133b: regulate proliferation, apoptosis, and PKM2 expression.
		Downregulated in HNOC: miR-133a, miR-99a, miR-194, **miR-133b**, miR-219, miR-100, miR-125b, **miR-26b**, **miR-138**, miR-149, miR-195, **miR-107**, **miR-139**.	
			
Kozaki et al., 2008 [27] Profiling for 148 miRNAs was carried out on 18 HNOC cell lines. One immortalized oral keratinocyte line was used as control.	qRT PCR	Upregulated in HNOC: miR-374, miR-340, miR-224, miR-10a, miR-140, miR-181a*, miR-146a, miR-126, **miR-31**, miR-9, miR-9*.	miR-137: regulates cell growth and CDK6 expression. miR-193a: regulates cell growth and E2F6 expression.
		Downregulated in HNOC: miR-27a, miR-34b, miR-34c, miR-203, miR-302c*, miR-23a, miR-27b, miR-34a, miR-215, miR-299, miR-330, miR-337, **miR-107**, **miR-133b**, **miR-138**, **miR-139**, miR-223, miR-204, miR-370, let-7d, miR-95, miR-302a, miR-367, let-7g, miR-23b, miR-128a, miR-148a, miR-155, miR-200c, miR-302b, miR-368, miR-122a, miR-371, let-7a, **miR-26b**, miR-30e-5p, miR-96, miR-125a, miR-132, miR-200b, miR-199b, miR-296, miR-373*, miR-137, miR-197, miR-193a, let-7e, miR-30d, miR-331, miR-342, miR-338, miR-199a, miR-372, miR-184	
